# A Case of Inversion of the Uterus

**Published:** 1847-01

**Authors:** E. Halley McCoy

**Affiliations:** Harrisville, Ohio


Art. IV.—*A Case of Inversion of the Uterus.* By E. Halley McCoy, M. D., of Harrisville, Ohio.

On the night of the 20th of last January I was summoned to visit Mrs. Carmon, æt 21, who had been delivered, two days previously, of her first child by a midwife. The patient was lying on her back, with feet drawn up in the bed; her respiration was diaphragmatic; skin hot and dry; pulse small, wiry, incompressible, and beating one hundred and fifteen in the minute; mouth dry; tongue covered with a dark brown fur. She had extreme abdominal tenderness; the lochia was suppressed, and she experienced continued bearing down pains. On making an examination *per vaginam*, I encountered a body which at first struck me as being the placenta, but on passing my finger upward around the tumor, I found the vagina reflected in the form of a ring around the pedicle of the tumor. I now became satisfied that I had an inverted womb to contend with. After explaining the case to the husband and friends, I introduced my hand in a conical form, indented the apex of the tumor, and gradually but perseveringly carried it up through the *os uteri*, entirely relieving the expulsive after pains. I ordered an infusion of chamomile flowers to be thrown into the vagina every two hours, together with fomentations of hops to the abdomen, and a powder containing grs. 5 pulv. antimonialis, gr. 2 calomel and gr. ½ opium; one to be taken every two hours. On my second visit, eighteen hours after reducing the inverted womb, I found a decided improvement in all the symptoms; the powders had acted freely on the bowels, bringing away copious, dark, bilious dejections; the abdominal tenderness was subsiding; the lochia had returned; the pulse had fallen to ninety and was soft and full; the skin was relaxed and moist, and the secretion of milk was established. In short, the patient required but little subsequent treatment, taking little else but a few ounces of elixir vitriol as a tonic, and at this time is able to attend to her domestic duties.

April 10th, 1846.

